# Heterogeneity in influenza seasonality and vaccine effectiveness in Australia, Chile, New Zealand and South Africa: early estimates of the 2019 influenza season

**DOI:** 10.2807/1560-7917.ES.2019.24.45.1900645

**Published:** 2019-11-07

**Authors:** Sheena G Sullivan, Carmen S Arriola, Judy Bocacao, Pamela Burgos, Patricia Bustos, Kylie S Carville, Allen C Cheng, Monique BM Chilver, Cheryl Cohen, Yi-Mo Deng, Nathalie El Omeiri, Rodrigo A Fasce, Orienka Hellferscee, Q Sue Huang, Cecilia Gonzalez, Lauren Jelley, Vivian KY Leung, Liza Lopez, Johanna M McAnerney, Andrea McNeill, Maria F Olivares, Heidi Peck, Viviana Sotomayor, Stefano Tempia, Natalia Vergara, Anne von Gottberg, Sibongile Walaza, Timothy Wood

**Affiliations:** 1 World Health Organization (WHO) Collaborating Centre for Reference and Research on Influenza, Royal Melbourne Hospital, and Doherty Department, University of Melbourne, at the Peter Doherty Institute for Infection and Immunity, Melbourne, Australia; 2 Influenza Division, Centers for Disease Control and Prevention, Atlanta, United States; 3 National Influenza Centre, Institute of Environmental Science and Research, Wellington, New Zealand; 4 Programa Nacional de Inmunizaciones, Ministerio de Salud, Santiago, Chile; 5 Sección de Virus Respiratorios y Exantematicos, Instituto de Salud Publica de Chile, Santiago, Chile; 6 Victorian Infectious Diseases Reference Laboratory, Royal Melbourne Hospital, at the Peter Doherty Institute for Infection and Immunity, Melbourne, Australia; 7 School of Public Health and Preventive Medicine, Monash University, Melbourne, Australia; 8 Department of Infectious Diseases, Alfred Health, and Central Clinical School, Monash University, Melbourne, Australia; 9 Discipline of General Practice, University of Adelaide, Adelaide, Australia; 10 National Institute for Communicable Diseases, Johannesburg, South Africa; 11 WHO Collaborating Centre for Reference and Research on Influenza, Royal Melbourne Hospital, at the Peter Doherty Institute for Reference and Research on Influenza, Melbourne, Australia; 12 Pan American Health Organization(PAHO)/WHO Regional Office for the Americas, Washington, United States; 13 Subdepartamento de Enfermedades Virales, Instituto de Salud Publica de Chile, Santiago, Chile; 14 Health Intelligence Team, Institute of Environmental Science and Research, Wellington, New Zealand; 15 Departamento de Epidemiologia, Ministerio de Salud, Santiago, Chile; 16 Influenza Program, Centers for Disease Control and Prevention, Pretoria, South Africa; 17 MassGenics, Duluth, United States

**Keywords:** influenza, vaccine effectiveness, southern hemisphere, sentinel surveillance, influenza vaccines

## Abstract

We compared 2019 influenza seasonality and vaccine effectiveness (VE) in four southern hemisphere countries: Australia, Chile, New Zealand and South Africa. Influenza seasons differed in timing, duration, intensity and predominant circulating viruses. VE estimates were also heterogeneous, with all-ages point estimates ranging from 7–70% (I^2^: 33%) for A(H1N1)pdm09, 4–57% (I^2^: 49%) for A(H3N2) and 29–66% (I^2^: 0%) for B. Caution should be applied when attempting to use southern hemisphere data to predict the northern hemisphere influenza season.

In Australia, Chile, New Zealand and South Africa, sentinel surveillance is conducted in primary care and/or hospitals to monitor the timing, intensity and impact of influenza seasons, and to estimate influenza vaccine effectiveness (VE). While the influenza epidemics of these four southern hemisphere countries often coincide, the type of epidemic experienced can vary. Nevertheless, the influenza season experienced in southern hemisphere countries has sometimes been interpreted as a forewarning to the northern hemisphere [1]. Here, we describe the heterogeneity experienced during the 2019 influenza season in these four countries and provide early VE estimates.

## Influenza surveillance systems

The sentinel surveillance systems used in this analysis are described in detail in the Table. For Australia, influenza-like illness (ILI) surveillance data came from the Australian Sentinel Practices Research Network (ASPREN), supplemented by the Victorian Sentinel Practice Influenza Network (VicSPIN) [2]. Hospital surveillance data were obtained from the Influenza Complications Alert Network (FluCAN) [3]. In Chile, severe acute respiratory infection (SARI) sentinel surveillance included seven sentinel hospitals distributed across six of 16 administrative regions [4]. In New Zealand, ILI surveillance leverages general practice-registered patients in all 20 district health boards, ca 540,000, while SARI surveillance includes four public hospitals in Auckland and Counties Manukau District Health Boards [5]. Syndromic surveillance data from South Africa came from outpatient presentations to a large private healthcare provider network, based on International Classification of Diseases (ICD-10) codes for pneumonia and influenza (J9-J11) [6,7]. Virological surveillance in South Africa was conducted through the Viral Watch network [8].

**Table:** Summary of key differences in case and exposure ascertainment for syndromic and virological surveillance and vaccine effectiveness estimation, four southern hemisphere countries, 2019 influenza season

Characteristic	Australia	Chile	New Zealand	South Africa
Source populations^a^	ILI: 394 GPs at sentinel general practices nationwide participate in syndromic ILI surveillance; 222 GPs participate in swab testing; 21 sentinel hospitals nation-wide	Seven sentinel hospitals in 6/16 regions	86 sentinel practices (ILI patients) in 20 district health boards and four hospitals (SARI patients)	Syndromic: a healthcare provider network Virological and VE: Sentinel general practices (ILI patients) in 6/9 regions
Period used for weekly rates	ILI: weeks 1–52 2019: weeks 1–39 Hospitals: weeks 14–44 2019: weeks 14–39	Weeks 1–52 2019: weeks 10–33	Weeks 18–39 2019: weeks 18–39	Weeks 1–52 2019: weeks 1–38
Clinical case definition	ILI: fever or history of fever AND cough, fatigue/malaise Hospitals: suspected influenza (not SARI)	SARI: history of fever, or measured fever of ≥ 38 C° AND cough AND onset within the last 10 days AND hospitalisation	ILI: acute respiratory illness with a history of fever or measured fever of ≥ 38 °C, AND cough, AND onset within the past 10 days SARI: as above, but requiring hospitalisation	ILI: measured fever (≥ 38 °C) or history of fever, cough, onset ≤ 10 days
Virological testing	ILI: Around 50% of patients are swabbed for testing by RT-PCR at SA Pathology, Adelaide or the NIC, Melbourne. Hospitals: RT-PCR testing done at each hospital. Sequencing performed by WHOCCRRI, Melbourne.	RT-PCR or direct immunofluorescence followed by RT-PCR-positive for pan-negative and influenza-positive specimens for subtyping. Testing and sequencing performed at NIC, Santiago.	RT-PCR testing at NIC, Wellington. Sequencing performed by WHOCCRRI, Melbourne.	RT-PCR testing by NIC, Johannesburg. Sequencing performed by WHOCCRRI, Melbourne or Worldwide Influenza Centre, Crick Institute, London.
Study period for VE estimation	ILI: 28 Apr 2019–9 Oct 2019 Hospitals: 1 Apr 2019–16 Aug 2019	SARI: 4 Mar 2019–18 Aug 2019	ILI and SARI: 29 Apr 2019–29 Sep 2019	ILI: 15 Apr 2019–18 Aug 2019
Cases/controls for VE estimates	ILI: test-positive cases vs test-negative controls Hospitals: test-positive cases; control are the next admitted test-negative patient (≤ 2 weeks)	Test-positive cases vs test-negative controls	Test-positive cases vs test-negative controls	Test-positive cases vs test-negative controls
Vaccination status ascertainment	Medical record, self-report or vaccination registry	Medical record or vaccination registries (no verbal reports)	Vaccination registry and self-report	Medical record or self-reported
Vaccination coverage among influenza-negative controls included in VE estimates^b^	Overall: 49% ILI; 47% hospitals Adults: 46% ILI; 41% hospitals Children: 26% ILI; 33% hospitals Elderly: 78% ILI; 73% hospitals	Overall: 61% SARI^c^ Adults: 41% SARI^c^ Children: 72% SARI^c^ Elderly: 64% SARI^c^	Overall: 26% ILI; 33% SARI Adults: 26% ILI; 36% SARI Children: 9% ILI Elderly: 70% ILI; 66% SARI	Overall: 11% ILI Adult: 11% ILI Children: 9% ILI Elderly: 35% ILI
Vaccines licensed	< 5 years: Flu Quadri Junior (Sanofi) < 65 years: Afluria Quad (Seqirus), FluQuadri (Sanofi) and Fluarix Tetra (GSK) ≥ 65 years: Fluad (Seqiris; trivalent with B/Yamagata component)	Influvac (Abbott) (inactivated subunit vaccine) TIV included a B/Victoria-lineage component	6–35 months: Fluarix Tetra (GSK) ≥ 3 years: FluQuadri (Sanofi), Influvac (Abbott) ≥ 5 years only: Afluria Quad (Seqiris)	Vaxigrip (Sanofi Pasteur) (inactivated split-virion vaccine) and Influvac (Abbott) (inactivated subunit vaccine) All TIV
Target groups for vaccination	Recommended for all. Free for pregnant women; people aged < 5 years or ≥ 65 years; Aboriginal and Torres Strait Islander peoples; people aged 5–64 years with chronic conditions.	Pregnant women from 13 weeks gestation; children aged 6–59 months, adults aged ≥ 65 years; poultry and pig farm workers; patients with chronic conditions aged 5– 64 years; carriers of some risk conditions; healthcare workers.	Pregnant women; people aged ≥ 65 years; people aged < 65 years with a medical condition that increases their risk of developing complications from influenza and the condition is specified in the Influenza Immunisation Programme eligibility criteria; children aged ≤ 4 years with previous hospitalisation for respiratory illness or with a history of significant respiratory illness.	Pregnant women at all stages of pregnancy, including the post-partum period; HIV-infected individuals; adults or children who are at high risk for influenza complications because of underlying medical conditions or who are receiving regular medical care for conditions such as chronic pulmonary disease; persons aged ≥ 65 years.

GP: general practice; GSK: Glaxo Smith Kline; ILI: influenza-like illness; NIC: National Influenza Centre; QIV: quadrivalent inactivated vaccine; SARI: severe acute respiratory illness; TIV: trivalent inactivated vaccine; VE: vaccine effectiveness; WHOCCRRI: World Health Organization Collaborating Centre for Reference and Research on Influenza.

^a^ Numbers are provided for 2019.

^b^ Children: 6 months–17 years of age; Adults: 18–64 years of age; Elderly: ≥ 65 years of age.

^c^ Only patients in a target group for vaccination are included in SARI surveillance in Chile so these numbers do not necessarily reflect coverage in the whole population.

## Seasonality

**Figure 1:**
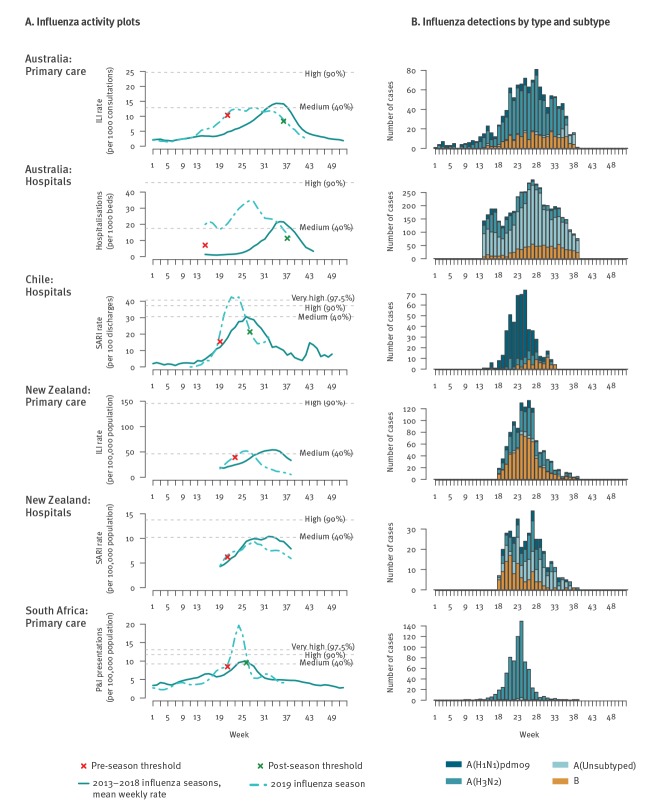
Influenza activity (A) and influenza detections (B) for Australia, Chile, New Zealand and South Africa, 2019 influenza season

ILI: influenza-like illness; P&I: pneumonia and influenza; SARI: severe acute respiratory infection.

Weekly 2019 influenza activity rates, e.g. ILI consultations per week, were plotted against the mean weekly rate for influenza seasons from 2013 to 2018. All rates were smoothed using a 3-week moving average. The moving epidemic method (MEM) package [9] in R software version 3.6.1 (R Foundation, Vienna, Austria) was used for calculating means and seasonal thresholds using default values to show the onset and intensity of the season (Figure 1A). The specifications used for the MEM may differ from published national surveillance reports. The onset and peak of the influenza season was at least 5 weeks early in Australia and 1 to 2 weeks early in Chile, New Zealand and South Africa. Activity was well above expected levels in South Africa and very high in Chile, but only reached moderate levels in Australia or New Zealand. The seasons experienced in Chile and South Africa were also much shorter in duration than in Australia and New Zealand.

Influenza activity plots (A) show the intensity of the 2019 influenza season compared with the average for 2013 to 2018. The point at which 2019 activity crossed baseline thresholds set by the prior 6 years’ data are marked with crosses. No post-season thresholds were estimated for New Zealand.

Influenza detections by type and subtype (B) for patients enrolled in hospital and primary care surveillance for VE estimation. The data used in vaccine effectiveness estimation are a subset restricted to those patients with complete information and recruited within the weeks used for estimation (Table).

## Virological data

Virological data are shown in Figure 1B and highlight the variation in predominant viruses circulating among countries. For example, while influenza A(H3N2) virus clearly predominated in South Africa and was detected at very high levels with the positivity reaching 80% during the peak period, the predominant virus in Chile was A(H1N1)pdm09. In New Zealand, both influenza A and B viruses were detected; however, their relative frequency differed between ILI and SARI surveillance, with B viruses detected among roughly half (51%; 604/1,179) of ILI patients but only a quarter (27%; 104/385) of SARI patients.

Genetic characterisation of selected viruses showed further differences among countries, although the number of samples characterised was small. Circulating A(H1N1)pdm09 viruses were similar, with most falling into subclade 6B.1A-P5 in Australia, New Zealand and Chile. Differences in the predominant circulating clade were observed for A(H3N2). Of 192 viruses sequenced in Australia, 186 were 3C.2a1b (3C.2a1b + 131K: n = 182; 3C.2a1b + 135K: n = 4), with just six 3C.3a. The majority of A(H3N2) viruses sequenced in New Zealand also clustered in clade 3C.2a1b. In Chile, of 31 viruses sequenced, 13 fell into the clade 3C.2a1b and 18 to 3C.3a. A limited selection of only 10 viruses from South Africa suggested co-circulation of 3C.2a1b + 131K, 3C.2a1b + 135K and 3C.3a viruses. For influenza B, nearly all viruses characterised in Australian primary care surveillance (107/108) and in New Zealand (167/169) were B/Victoria lineage viruses, while all 11 influenza B viruses characterised in Chile were B/Yamagata.

## Vaccine effectiveness estimation

**Figure 2:**
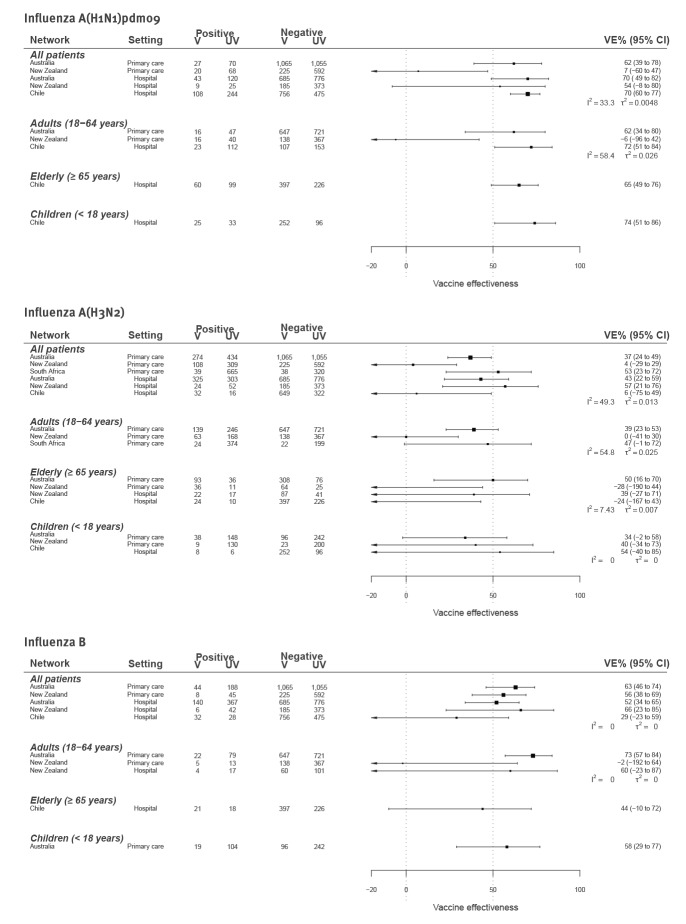
Early vaccine effectiveness estimates against influenza A(H1N1)pdm09, A(H3N2) and B by age group and setting, Australia, Chile, New Zealand and South Africa, 2019 influenza season

CI: compatibility interval; V: vaccinated; UV: unvaccinated; VE: vaccine effectiveness.

The virological data depicted in Figure 1B formed the basis for VE estimation. All systems followed a test-negative design, where the odds ratio (OR) comparing the odds of vaccination among test-positive cases vs test-negative controls was used to derive VE, i.e. VE = (1−OR_adj_)×100% [10]. Estimates were made separately for each country, virus and age group, incorporating covariates considered important by each site (Figure 2). The heterogeneity among estimates within each virus/age group combination was measured by I^2^ and τ^2^ [11]. All networks were able to provide data for the A(H3N2) VE. Too few A(H1N1)pdm09 and B cases were detected in South Africa to enable VE estimation.

I^2^ and τ^2^are shown for measures of heterogeneity.

Estimates for Chile only include patients in a target group for vaccination; Australia used adjuvanted TIV for individuals ≥ 65 years of age.

Covariate adjustment: Australia primary care estimates adjusted for week (restricted cubic spline) and age group (where appropriate); Australia hospital estimates adjusted for age group, comorbidities, indigenous ethnicity and pregnancy; Chile estimates adjusted for age, month of symptom onset and pre-existing conditions; New Zealand estimates adjusted for age group; South Africa estimates adjusted for seasonality and age.

For A(H1N1)pdm09, heterogeneity was low overall (I^2^: 22%). For adults, although heterogeneity was not high (I^2^: 58%), VE estimates ranged from −6% (95% compatibility interval (CI): −96 to 42) in New Zealand to 72% (95% CI: 51–84) among people in a target group for vaccination in Chile. Only Chile was able to provide VE estimates for children (65%; 95% CI: 49–76) and elderly, i.e. adults aged ≥ 65 years (74%; 95% CI: 51–86).

For A(H3N2), heterogeneity was moderate overall (I^2^: 49%), but higher for adults (I^2^: 59%). In Australia, South Africa and New Zealand hospitals, VE point estimates ranged from 34% to 57% across age groups; however, in Chile and New Zealand primary care, estimates were often close to or beyond the null.

For influenza B, heterogeneity was low overall (I^2^: 0%), despite differences in the predominant lineage and the use of trivalent vaccine in Chile but quadrivalent in New Zealand and Australia. Overall VE was lowest in Chile (29%; 95% CI: −23 to 59). Here, the B component for trivalent vaccines included a B/Victoria-like virus, but most viruses circulating were B/Yamagata thereby suggesting this low VE may be attributable to lineage mismatch. Only one VE estimate was available for elderly adults (Chile: 44%; 95% CI: −10 to 72) and children (Australia: 55%; 95% CI: 20–76).

## Discussion

We have shown that within countries of the southern hemisphere, the timing, duration and intensity of the influenza seasons, the predominant circulating viruses, and VE all varied in the 2019 influenza season, even between neighbouring countries such as Australia and New Zealand. Similar observations have been reported from Europe [9]. Thus, it appears that activity in one country is not indicative of activity in another country, even when influenza seasons are contemporaneous.

The early VE estimates for the 2019 influenza season in the southern hemisphere presented here were highest for influenza A(H1N1)pdm09 and lowest for A(H3N2). Early estimates often approximate final estimates [12]. However, the utility of these estimates for the northern hemisphere may be limited because the 2019 southern hemisphere vaccine differed from the 2019/20 northern hemisphere formulation in three of four components, A(H1N1)pdm09, A(H3N2) and B/Victoria. Nevertheless, these estimates or earlier versions of them were included with other data reviewed at the WHO Consultation and Information Meeting on the Composition of Influenza Virus Vaccines for Use in the 2020 Southern Hemisphere Influenza Season during 23–26 September 2019 in Geneva and provided a general impression of the performance of the 2019 vaccine.

While heterogeneity in our VE estimates did not exceed an I^2^ of 60%, with so few studies, the sensitivity of statistical tests to detect heterogeneity is probably limited. This is exemplified by the I^2^ of 0% for influenza B estimates among adults despite differences in VE point estimates of 75 percentage points (Figure 2). Thus, low heterogeneity statistics do not alleviate concerns about how to interpret discrepant VE point estimates.

There are many potential sources for this heterogeneity that affect not only the VE estimates, but interpretation of weekly activity rates. First, with random sampling, we should not expect estimates to be the same [13]. Second, when samples are small they may be vulnerable to statistical biases, such as sparse data bias, and bias due to measurement errors may be more profound [14]. Third, there were many differences in study design (Table). Case ascertainment differed; for example, a SARI case definition was used in New Zealand and Chile, but not in Australian hospital surveillance. Exposure ascertainment also differed, with varying availability of registries to verify vaccination status and the use of different vaccines. In particular, the adjuvanted vaccines used among Australians ≥ 65 years of age might be expected to yield higher VE than standard vaccines [15]. Fourth, vaccine coverage varied (Table). Low vaccination coverage, as observed in South Africa, affects power and precision and can exacerbate the bias induced by measurement errors. Higher coverage, as seen in Chile and among elderly patients in New Zealand and Australia, may mean that many more people in the sample are repeat vaccinees. Repeat vaccination may negatively impact VE and could result in lower VE estimates in highly vaccinated populations [16]. Finally, although only limited virological data were available, we observed differences in circulating A(H3N2) virus clades and B lineages. This may impact both seasonality and VE, particularly as most A(H3N2) viruses sequenced appeared to be in different clades from the vaccine virus (3C.2a2). Notably, most A(H3N2) viruses were also in different genetic groups from the 2019/20 northern hemisphere vaccine (3C.3a).

In conclusion, we have attempted to briefly summarise and interpret the 2019 influenza season in four southern hemisphere countries and have presented early VE estimates. We observed substantial variation in available data on influenza seasonality and VE within the southern hemisphere in 2019, which is unsurprising given the many differences in surveillance among these countries. Caution should be applied when attempting to infer the impending northern hemisphere influenza season based on these observations.
